# Fast-GBS: a new pipeline for the efficient and highly accurate calling of SNPs from genotyping-by-sequencing data

**DOI:** 10.1186/s12859-016-1431-9

**Published:** 2017-01-03

**Authors:** Davoud Torkamaneh, Jérôme Laroche, Maxime Bastien, Amina Abed, François Belzile

**Affiliations:** 1 Département de Phytologie, Université Laval, Quebec City, QC Canada; 2 Institut de Biologie Intégrative et des Systèmes (IBIS), Université Laval, Quebec City, QC Canada

**Keywords:** NGS, GBS, Bioinformatics pipeline, SNP, Genotype accuracy

## Abstract

**Background:**

Next-generation sequencing (NGS) technologies have accelerated considerably the investigation into the composition of genomes and their functions. Genotyping-by-sequencing (GBS) is a genotyping approach that makes use of NGS to rapidly and economically scan a genome. It has been shown to allow the simultaneous discovery and genotyping of thousands to millions of SNPs across a wide range of species. For most users, the main challenge in GBS is the bioinformatics analysis of the large amount of sequence information derived from sequencing GBS libraries in view of calling alleles at SNP loci. Herein we describe a new GBS bioinformatics pipeline, Fast-GBS, designed to provide highly accurate genotyping, to require modest computing resources and to offer ease of use.

**Results:**

Fast-GBS is built upon standard bioinformatics language and file formats, is capable of handling data from different sequencing platforms, is capable of detecting different kinds of variants (SNPs, MNPs, and Indels). To illustrate its performance, we called variants in three collections of samples (soybean, barley, and potato) that cover a range of different genome sizes, levels of genome complexity, and ploidy. Within these small sets of samples, we called 35 k, 32 k and 38 k SNPs for soybean, barley and potato, respectively. To assess genotype accuracy, we compared these GBS-derived SNP genotypes with independent data sets obtained from whole-genome sequencing or SNP arrays. This analysis yielded estimated accuracies of 98.7, 95.2, and 94% for soybean, barley, and potato, respectively.

**Conclusions:**

We conclude that Fast-GBS provides a highly efficient and reliable tool for calling SNPs from GBS data.

## Background

Currently, genomics lies at the heart of an extraordinary number of discoveries, innovations and applications. This revolution is a direct result of the rise of next-generation sequencing (NGS) technologies [1–4]. In the area of genotyping, the combination of NGS and reduced representation methods, which focus the sequencing effort on a small subset of the genome, has made it possible to simultaneously perform genome-wide single nucleotide polymorphism (SNP) discovery and genotyping in a single step even in species with large genomes [5]. This has facilitated greatly the genotyping of very large numbers of SNPs using a number of related methods (e.g. CRoPS, RAD-seq, GBS, double-digest RAD-seq, and 2bRAD) [6–11]. These various methods make it possible to study important questions in molecular breeding, population genetics, ecological genetics and evolution using thousands to millions of genetic markers in a wide array of species [5]. Genotyping-by-sequencing (GBS) is a particularly attractive complexity reduction method that offers a simple, robust, low-cost, and high-throughput method for genotyping in both model and non-model species [8].

Advanced sequencing technologies (NGS) have reduced both the cost and the time required to generate sequence data. The efficient and accurate computational processing, variant and genotype calling of large-scale NGS data is the new bottleneck in genomics. To meet this need, numerous bioinformatics pipelines have been developed [12–16] and all need to accomplish a similar set of steps such as: 1) acquiring raw sequence data, 2) demultiplexing pooled sequence read data, 3) filtering out low-quality reads, 4) assembling or aligning reads, and finally 5) discovering polymorphic loci and inferring actual genotypes at these loci. Each step represents a set of specific challenges and ambiguities. Generally, various genomic characteristics such as the number of detected variants, the complexity of the genome, the degree of heterozygosity, the proportion of repetitive sequences throughout the whole genome, the level of polymorphism and divergence among populations can contribute to these challenges [12]. On the other hand, technical factors such as DNA quality, the degree of sample multiplexing, the total number of reads per sample, the length of reads, and the sequencing error rate interact with these biological factors [17–19]. Therefore, it is necessary to select appropriate parameters such as the required depth of coverage, the quality of read mapping or the allowable degree of divergence for successful mapping. Finally, because of these two different sources of variation (biological and technical) in GBS data, the optimal parameters need to be adjusted for each species and desired marker coverage and throughput.

Conventionally, bioinformatics pipelines for handling GBS data are categorized in two groups: *de novo*-based and reference-based. In the presence of a reference genome, the reads from reduced-representation sequencing can be mapped to the reference genome and SNPs can be called [12, 20]. Up to now, several reference-based GBS analysis pipelines have been developed. The most widely used reference-based GBS analysis pipelines are: TASSEL-GBS (v1 and v2), Stacks, and IGST [13–15, 21]. But when a reference genome is not available, pairs of nearly identical reads (presumed to represent alternative alleles at a locus) need to be identified. The most highly used pipelines for such a *de novo*-based approach are UNEAK and Stacks [15, 16].

Herein, we describe a new reference-based pipeline, Fast-GBS, and we benchmark the pipeline based upon a large-scale, species-wide analysis of soybean, barley and potato. It is easy to use with various species, in different contexts, and provides an analysis platform that can be run with different types of sequencing data and modest computational resources.

### Test datasets

To test the performance of Fast-GBS, we used existing sequence datasets for panels of 24 unrelated accessions/clones for three species covering a range of genomic situations: soybean [22], barley [Abed et al., unpublished], and potato [Bastien et al., unpublished]. Table 1 shows the species which we used in this study. These vary in terms of their ploidy, genome size and mode of reproduction (which relates to the expected zygosity). We used sequence datasets composed of 24 samples for each species.

**Table 1:** List of species genotyped using a GBS approach and analyzed using Fast-GBS. For the three different species used in this work, relevant characteristics (ploidy, genome size, reproduction mode and chromosome number) influencing GBS analysis are shown

Name	Species	Ploidy	Genome size (Mb)	Mode of reproduction	Number of chromosomes
Soybean	*Glycine max*	Paleotetraploid	1,100	Selfing	20 [40]
Barley	*Hordeum vulgare*	Diploid	5,300	Selfing	7 [41]
Potato	*Solanum tuberosum*	Autotetraploid	844	Clonal	12 [42]

### Genotype validation

To estimate genotype accuracy for Fast-GBS calls, we compared the called SNPs with independently derived genotypic data resulting from either whole-genome resequencing (soybean and barley) or genotyping on a SNP array (potato) for the same samples. For soybean, we compared the GBS-called SNPs with whole genome resequencing data for the same 24 samples. In the case of barley, GBS-derived genotypic data for one of the 24 barley samples (cv. Morex) was compared to the barley reference genome produced using this same cultivar. For potato, we compared the GBS-derived genotypes with those obtained for the same 24 samples at a set of 122 SNPs that were in common with the SolCAP Infinium Chip (8.3 k SNPs) [23].

## Implementation

**Fig. 1:**
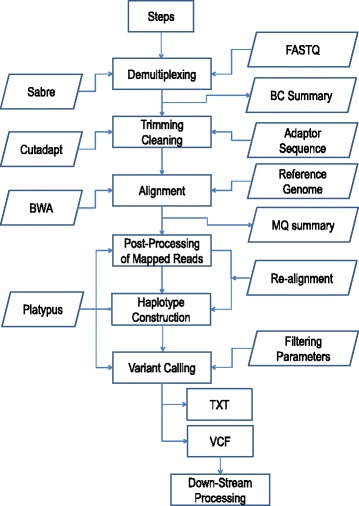
Schematic representation of the analytical steps in the Fast-GBS pipeline. The main steps in the analytical process are indicated in the central portion of the diagram, while the different software tools used are indicated to the left and inputs and outputs of each step to the right

The Fast-GBS analysis pipeline was developed by integrating public packages with internally developed tools. The public packages include Sabre (demultiplexing) [24], Cutadapt (read trimming and cleaning) [25], BWA (read mapping) [26], SAMtools (file conversion and indexing) [27], and Platypus (post-processing of reads, haplotype construction and variant calling) [28]. Fast-GBS functions and software tools are presented in Fig. 1.

### Creating directory structure

We developed a Bash script to create the directory structure before running the Fast-GBS pipeline. This command line creates the directories for data (FASTQ files), barcodes (key file), reference genome, and results (Fast-GBS outputs).

### Input

The input data are sequenced DNA fragments from any restriction enzyme–based GBS protocol. Fast-GBS handles raw sequencing data in FASTQ format.

### Preparing the parameter file

The parameter file is a text file containing key information about the analysis including the path to the FASTQ files, barcodes and reference genome. It also contains information about the type of sequence (paired or single-end), the adaptor sequence and the sequencing technology. In this file we can define critical filtering options such as the minimal quality scores for reads, minimal number of reads required to call a genotype, and maximal amount of missing data allowed. Number of CPU, names of output files are also defined in this file. This file comes with the Fast-GBS pipeline.

### Data demultiplexing

The cost efficiency of GBS is partly due to the multiplexing of samples and the resulting pooled reads will need to be demultiplexed prior to SNP calling. Fast-GBS uses Sabre [24] to demultiplex barcoded reads into separate files. It simply compares the provided barcodes with the 5′ end of each read and separates the reads into the appropriate barcode files after having clipped the barcode from the read. If a read does not have a recognized barcode, it is put into an “unknown” file. Sabre also has an option (-m) to allow mismatches within barcodes. Sabre supports gzipped input files. After demultiplexing, Sabre outputs a BC summary log file of how many reads went into each barcode file.

### Trimming and cleaning

After demultiplexing, Fast-GBS uses Cutadapt to find and remove adapter sequences, primers, and other types of unwanted sequence from high-throughput sequencing reads [25].

### Read mapping algorithms

Fast-GBS uses the MEM (maximal exact matches) algorithm implemented in BWA that works by seeding alignments and then extending seeds with the Smith-Waterman (SW) algorithm using an affine gap penalty [26]. This algorithm can perform local alignment for reads of 70 bp up to 1Mbp. This algorithm can perform parallel alignment, thus markedly increasing the speed of the analysis. The ability to align reads of variable size allows the use of data obtained using different sequencing platforms (Illumina, Ion Torrent, etc). Aligned reads may be gapped to allow for Indels.

### Post-processing of mapped reads

After initial alignment, the mapped reads are further processed by Platypus [28] in order to improve the sensitivity and specificity of variant calling. This post-processing seeks to improve the quality of mapping by performing a re-examination of poorly mapped reads and reads mapping to multiple locations. Platypus classifies poorly mapped reads in three categories: 1) reads with numerous mismatches (high level of sequencing errors), 2) reads mapping to multiple locations in the genome, and 3) any remaining linker or adaptor sequences (causing poor mapping). Variants called using such potentially incorrectly mapped reads (see next step) are highlighted using a BadReads flag.

### Haplotype construction and variant calling

In Fast-GBS, variants are called using Platypus. Unlike alignment-based variant callers which focus on a single variant type (SNP or indel), Platypus uses multi-sample variant calling that helps to exploit information from multiple samples to call variants that may not look reliable in a single sample. This approach decreases the errors around indels and larger variants (MNPs). At first, the local assembler looks at a small window (~few kb) at a time and uses all the reads in the window to generate a colored de Bruijn graph, then using all candidate variants, it generates an exhaustive list of haplotypes. Candidate haplotypes are generated by clustering the candidate alleles across windows. Haplotype frequencies are estimated by the expectation-maximization (EM) algorithm. Then variants are called using the estimated haplotype frequencies. This approach works on the local haplotype level rather than on the level of individual variants and does well on highly divergent regions. This also decreases computational requirements.

### Variant and individual-level filtering

Platypus was originally designed and used to detect variants in human, mouse, rat and chimpanzee samples. To optimize Platypus options in the context of the analysis of GBS-derived single-end reads, we modified several options (see [29] for details of Platypus options). Some of the filters used in Fast-GBS variant calling steps are: number of reads (NR) per locus (default = 2), mapping quality score of reads to call a variant (MQ ≥ 10), minimum base quality (default = 10), MNPs distance (minFlank = 5), and maximum missing data (MaxMD) allowed (default ≤ 80%). See Fast-GBS user manual for a full description of all filtering options.

### Output data

The main output file of Fast-GBS is a.vcf file [30] containing detailed information on each of the variants. In addition, Fast-GBS also generates a simple text file containing only the genotypic data. The Fast-GBS log file contains the completed steps of the pipeline as it is running. In cases where an error occurs and prematurely terminates the running of the pipeline, the log file shows the step at which the analysis stopped. An analysis can be started at any point on the existing intermediate files simply by creating a log file in which the previously completed steps are listed. Fast-GBS will re-initiate the analysis starting from that point onwards.

## Results and Discussion

### Performance of Fast-GBS

To assess the performance of the Fast-GBS analysis pipeline, we used it to analyze existing GBS-derived read data from sets of 24 soybean, barley, and potato samples. Table 2 presents a summary of this analysis. As can be seen, a total of 35 k SNPs were called using 42 M 100-bp Illumina reads on *Ape*KI-digested DNA from 24 different soybean lines. Similarly, for barley, 32 k SNPs were successfully called from 72 M Ion Torrent reads (50–150 bp in length) derived from a 24-plex *Msp*I/*Pst*I library. Finally, in potato, 38 k SNPs were obtained from sequencing a 24-plex *Msp*I/*Pst*I library (43 million 100-bp Illumina reads).

**Table 2:** Number of variants detected among 24 soybean, barley, and potato samples. The sequencing platform, number of reads, filtering options, and genotype accuracy for each dataset are also provided

				Filtering options^a^				
Name	Sequencing platform	Restriction enzyme	Number of reads	minNR	MinMAF	MaxMD (%)	Number of variants	Accuracy (%)
Soybean	Illumina	*Ape*KI	42 M	2	0.04	80	35 k	98.7
Barley	Ion Torrent	*Msp*I/*Pst*I	72 M	2	0.04	80	32 k	95.2
Potato	Illumina	*Msp*I/*Pst*I	43 M	11	0.04	20	38 k	94.0

^a^Filtering options: *minNR* minimum number of reads to call a variant (depth), *MinMAF* minimum minor allele frequency, and *MaxMD* maximum missing data allowed

GBS was originally demonstrated for soybean by Sonah et al [21] using the IGST pipeline. Using 8 diverse soybean lines, they called ~10 k SNPs. Later work by the same group lead to the calling of 45 k SNPs on a large collection of 304 soybean lines for the purpose of conducting a GWAS study [31]. Analysis of this dataset using IGST took four days while the same analysis using Fast-GBS at the same sample size and condition took only 11 h and called ~60 k SNPs (data not shown). As can be seen Fast-GBS present a high level of performance for soybean samples.

Barley has one of the larger genomes (>5 Gb) among cultivated plant species. Because of the huge size and high level of complexity of its genome, complexity reduction is highly recommended in barley, an important crop species for which a draft genome has been published [32]. Mascher et al [33] genotyped 94 barley RIL lines using GBS (*Msp*I/*Pst*I-digested library) and they called 34 k and 19 k SNPs using either the reference genome (with SAMtools) or a *de novo* pipeline (TASSEL), respectively. In this study we used Fast-GBS for SNP calling in barley and, as can be seen in Table 2, Fast-GBS called 32 k SNPs for a small number of samples (24). This showed the capability of Fast-GBS to run with large and complex genomes.

Because of the high level of ploidy and heterozygosity, potato is a challenging species for genotyping. The most often used method for genotyping in potato is a SNP array. Two SNP arrays have been developed so far, the SolCAP 8 k and 20 k arrays [23, 34, 35]. Recently, Endelman [36], genotyped 96 F2 diploid potato samples using GBS. Using an R-based bioinformatics pipeline to filter the GBS variants, they identified 11 k SNPs. In this study, we called 38 k SNPs from 24 samples which had also been genotyped using the SolCAP 8 k SNP array. Using a simplified genotyping mode (“diploid mode”) in which only three genotypic classes were distinguished (0/0, 0/1 and 1/1), 5.5 k SNPs on this array had been found to be polymorphic among this set of 24 potato samples [37]. As can be seen in Table 2, using Fast-GBS to call SNPs in an equivalent diploid mode, we called almost seven times more polymorphisms than using a SNP array (38 k vs 5.5 k SNPs).

### Validation of Fast-GBS data

An important aspect to consider for any variant calling tool is the accuracy of called genotypes. In this study, we estimated the accuracy of genotypes called by Fast-GBS (Table 2) by comparing them to the “true” genotypes (obtained from either whole-genome resequencing or SNP array data). For soybean, for all 24 samples, we compared the SNP genotypes called by Fast-GBS to the genotypes assigned to the same loci following whole-genome sequencing. We found a very high level of concordance, as almost all genotypes (98.7%) proved identical. For barley, we compared the SNP genotypes called by Fast-GBS with the true genotypes for one of the 24 lines (cv. Morex), the only one for which we had whole genome sequencing data. Again, a high degree of agreement between the two datasets (97%) was obtained. Finally, for potato, we used data obtained on the SolCAP 8 k Infinium Chip for the same 24 samples used to perform GBS. These two datasets shared 122 SNP loci. In our initial comparison, only 87.7% were in agreement. When we examined the proportion of concordant calls, we discovered that more than 50% of all discordant calls came from only three samples and the degree of discordance in these was so great that it suggested we were not comparing the same clones. After removing these outliers from the analysis, 94% of genotypes called by Fast-GBS and the SNP array were in agreement in the remaining 21 clones. We conclude that Fast-GBS can accurately call SNPs in species with different characteristics (genome size, ploidy, zygosity).

### Flexibility to run different sequencing platforms

In this study, to assess the performance of Fast-GBS, we used both Illumina and Ion Torrent reads. Soybean and potato samples were sequenced using an Illumina Hiseq platform and barley samples on an Ion Torrent (Proton) platform. Typically for GBS, Illumina sequencing generates reads of uniform length (100 bp), while Ion Torrent reads are in 50 to 150 bp. Ion Torrent sequencing usually leads to a higher rate of sequencing errors [38, 39]. Thus, it is preferable for an analytical pipeline to be versatile and capable of using reads derived from either technology (or new technologies in development). Most GBS bioinformatics pipelines are able to proceed with Ion Torrent reads, but often need to be modified to be suitable for this type of read data. TASSEL, UNEAK, and Stacks generate tags of a fixed length (e.g. 64 bp). This will lead to an important loss of sequence information and can lead to inaccurate or ambiguous mapping of reads. Also, because of the increased amount of sequencing errors, these pipelines can generate false tags which produce false SNPs. As shown above, Fast-GBS proved the capacity of accurately proceed maximum SNP calling using reads obtained from both sequencing platforms (Ion Proton and Illumina).

## Conclusions

GBS provides an extremely powerful and versatile tool for identifying and calling genetic markers to be used by researchers working in numerous species and fields of study. This genotyping approach, like all applications based on NGS, generates a huge amount of raw data. These data need to be analyzed as quickly and efficiently as possible, all the while yielding SNP data that is highly accurate. Fast-GBS showed itself to be a powerful pipeline to generate large numbers of highly accurate SNPs using sequence read data obtained from different sequencing platforms and diverse species characterized by different levels of ploidy, zygosity, and genome complexity. By combining efficiency and accuracy in this way, Fast-GSB constitutes a useful tool for a broad array of users in different research communities.

## Availability and requirements

**Project name:** Fast-GBS

**Project home page:** https://bitbucket.org/jerlar73/fast-gbs

**Operating system:** Linux

**Programming language:** Bash and Python

**License:** GNU GPL v

**Any restrictions to use by non-academics:** No

Sequence data are available in NCBI Sequence Read Archive (SRA) with the SRP# Study accession, SRP059747.

## Abbreviations

CRoPS: Complexity reduction of polymorphic sequences
GBS: Genotyping-by-sequencing
Indel: Insertion-deletion
MaxMD: Maximum missing data
MinMAF: Minimum minor allele frequency
minNR: Minimum number of reads
MNP: Multiple nucleotide polymorphism
MQ: Mapping quality
NGS: Next-generation sequencing
RAD-seq: Restriction site associated DNA sequencing
SNP: Single nucleotide polymorphism

## References

[1] Metzker ML. Sequencing technologies - the next generation. Nat Rev Genet. 2010:31–46. doi:10.1038/nrg2626 10.1038/nrg2626 19997069

[2] Edwards D, Batley J, Snowdon RJ (2013). Accessing complex crop genomes with next-generation sequencing. Theor Appl Genet.

[3] Kilpinen H, Barrett JC (2013). How next-generation sequencing is transforming complex disease genetics. Trends Genet.

[4] Kumar S, Banks TW, Cloutier S (2012). SNP discovery through next-generation sequencing and its applications. Int J Plant Genom.

[5] Davey JW, Hohenlohe PA, Etter PD, Boone JQ, Catchen JM, Blaxter ML (2011). Genome-wide genetic marker discovery and genotyping using next-generation sequencing. Nature.

[6] van Orsouw NJ, Hogers RCJ, Janssen A, Yalcin F, Snoeijers S, Verstege E (2007). Complexity reduction of polymorphic sequences (CRoPS): a novel approach for large-scale polymorphism discovery in complex genomes. PLoS One.

[7] Etter PD, Atwood TS, Currey MC, Shiver AL, Lewis ZA (2007). Rapid SNP Discovery and Genetic Mapping Using Sequenced RAD Markers. PLoS One.

[8] Elshire RJ, Glaubitz JC, Sun Q, Poland JA, Kawamoto K, Buckler ES (2011). A robust, simple genotyping-by-sequencing (GBS) approach for high diversity species. PLoS One.

[9] Etter PD, Bassham S, Hohenlohe PA, Johnson EA, Cresko WA (2001). SNP discovery and genotyping for evolutionary genetics using RAD sequencing. Methods Mol Biol.

[10] Peterson BK, Weber JN, Kay EH, Fisher HS, Hoekstra HE (2012). Double digest radseq: an inexpensive method for de novo SNP discovery and genotyping in model and nonmodel species. PLoS One.

[11] Wang S, Meyer E, McKay JK, Matz MV (2012). 2b-RAD: a simple and flexible method for genome-wide genotyping. Nat Methods.

[12] Nielsen R, Paul JS, Albrechtsen A, Song YS (2011). Genotype and SNP calling from next-generation sequencing data. Nature Rev Genet.

[13] Bradbury PJ, Zhang Z, Kroon DE, Casstevens TM, Ramdoss Y, Buckler ES (2007). TASSEL: software for association mapping of complex traits in diverse samples. Bioinformatics.

[14] Glaubitz JC, Casstevens TM, Lu F, Harriman J, Elshire RJ, Sun Q (2014). TASSEL-GBS: A High Capacity Genotyping by Sequencing Analysis Pipeline. PLoS One.

[15] Catchen J, Hohenlohe PA, Bassham S, Amores A, Cresko WA (2013). Stacks: an analysis tool set for population genomics. Mol Ecol.

[16] Lu F, Lipka AE, Glaubitz J, Elshire R, Cherney JH (2013). Switchgrass Genomic Diversity, Ploidy, and Evolution: Novel Insights from a Network-Based SNP Discovery Protocol. PLoS Genet.

[17] Gompert Z, Forister ML, Fordyce JA, Nice CC, Williamson RJ, Buerkle CA (2010). Bayesian analysis of molecular variance in pyrosequences quantifies population genetic structure across the genome of Lycaeides butterflies. Mol Ecol.

[18] Lynch M (2009). Estimation of allele frequencies from high coverage genome-sequencing projects. Genetics.

[19] Hohenlohe PA, Phillips PC, Cresko WA. Using population genomics to detect selection in natural populations: key concepts and methodological considerations. Int J Plant Sci. 2010a;171:1059–1071 10.1086/656306 PMC3016716 21218185

[20] Li H, Durbin R (2009). Fast and accurate short read alignment with Burrows-Wheeler transform. Bioinformatics.

[21] Sonah H, Bastien M, Iquira E, Tardivel A, Legare G (2013). An Improved Genotyping by Sequencing (GBS) Approach Offering Increased Versatility and Efficiency of SNP Discovery and Genotyping. PLoS One.

[22] Torkamaneh D, Belzile F (2015). Scanning and Filling: Ultra-Dense SNP Genotyping Combining Genotyping-By-Sequencing, SNP Array and Whole-Genome Resequencing Data. PLoS One.

[23] Felcher KJ, Coombs JJ, Massa AN, Hansey CN, Hamilton JP (2012). Integration of Two Diploid Potato Linkage Maps with the Potato Genome Sequence. PLoS One.

[24] Sabre-barcode-demultiplexing: https://github.com/najoshi/sabre. Accessed 27 Sept 2013.

[25] Martin M. Cutadapt removes adapter sequences from high-throughput sequencing reads. EMBnet J. 2011. doi:http://dx.doi.org/10.14806/ej.17.1.200.

[26] Li H. and Durbin R. Fast and accurate long-read alignment with Burrows-Wheeler Transform. Bioinformatics. 2010. Epub. [PMID: 20080505] 10.1093/bioinformatics/btp698 PMC2828108 20080505

[27] Li H (2011). A statistical framework for SNP calling, mutation discovery, association mapping and population genetical parameter estimation from sequencing data. Bioinformatics.

[28] Rimmer A, Phan H, Mathieson I, Iqbal Z, Twigg SRF (2014). Integrating mapping-, assembly- and haplotype-based approaches for calling variants in clinical sequencing applications. Nat Genet.

[29] Platypus-Sapelo: https://wiki.gacrc.uga.edu/wiki/Platypus-Sapelo. Accessed 9 Dec 2015.

[30] Danecek P, Auton A, Abecasis G, Albers CA, Banks E (2011). The Variant Call Format and VCFtools. Bioinformatics.

[31] Sonah H, O’Donoughue L, Cober E, Rajcan I, Belzile F (2014). Identification of loci governing eight agronomic traits using a GBS-GWAS approach and validation by QTL mapping in soya bean. Plant Biotechnol J.

[32] The International Barley Genome Sequencing Consortium (2012). A physical, genetic and functional sequence assembly of the barley genome. Nature.

[33] Mascher M, Wu S, Amand PS, Stein N, Poland J (2013). Application of Genotyping-by-Sequencing on Semiconductor Sequencing Platforms: A Comparison of Genetic and Reference-Based Marker Ordering in Barley. PLoS One.

[34] Peter GV, Uitdewilligen JGAML, Voorrips E, Visser RGF, van Eck HJ (2015). Development and analysis of a 20 K SNP array for potato (*Solanum tuberosum*): an insight into the breeding history. Theor Appl Genet.

[35] Prashar A, Hornyik C, Young V, McLean K, Sharma SK, Dale MF, Bryan GJ (2014). Construction of a dense SNP map of a highly heterozygous diploid potato population and QTL analysis of tuber shape and eye depth. Theor Appl Genet.

[36] Endelman J (2015). Genotyping-By-Sequencing of a Diploid Potato F2 Population.

[37] Hirsch CN, Hirsch CD, Felcher K, Coombs J, Zarka D, Van Deynze A, De Jong W, Veilleux RE, Jansky S, Bethke P. Retrospective view of North American potato (Solanum tuberosum L.) breeding in the 20th and 21st centuries. 2013. G3 3:1003–1013. doi:10.1534/G3.113.005595. 10.1534/g3.113.005595 PMC3689798 23589519

[38] Golan D, Medvedev P (2013). Using state machines to model the Ion Torrent sequencing process and to improve read error rates. Bioinformatics.

[39] Bragg LM, Stone G, Butler MK, Hugenholtz P, Tyson GW (2013). Shining a Light on Dark Sequencing: Characterising Errors in Ion Torrent PGM Data. PLoS Comput Biol.

[40] Stacey G. Genetics and Genomics of Soybean. New York: Springer; 2008. ISBN978-0-387-72299-3.

[41] Zhang G, Li C, Liu X. Advance in Barley Sciences. Dordrecht, Heidelberg, New York: Springer; 2013. ISBN 978-94-007-4682-4.

[42] Bradeen JM, Kole C. Genetics, Genomics and Breeding of Potato. NH: Enfield, CRC Press; 2011. ISBN 9781578087150.

